# Deubiquitinase PSMD7 facilitates pancreatic cancer progression through activating Nocth1 pathway via modifying SOX2 degradation

**DOI:** 10.1186/s13578-024-01213-9

**Published:** 2024-03-17

**Authors:** Chen Luo, Yi Yu, Jinfeng Zhu, Leifeng Chen, Dan Li, Xingyu Peng, Zitao Liu, Qing Li, Qing Cao, Kai Huang, Rongfa Yuan

**Affiliations:** 1 Department of General Surgery, The Second Affiliated Hospital of Nanchang University, Nanchang, Jiangxi Province 330006 China; 2 Department of General Surgery, The First Affiliated Hospital, Jiangxi Medical College, Nanchang University, Nanchang, Jiangxi Province 330006 China; 3 Department of Urology Surgery, The Second Affiliated Hospital of Nanchang University, Nanchang, Jiangxi Province 330006 China; 4 Hunan Provincial Key Laboratory of the Research and Development of Novel Pharmaceutical Preparations, Changsha Medical University, Changsha, Hunan Province 410219 China; 5 Department of Gastroenterology, The Second Affiliated Hospital of Nanchang University, Nanchang, Jiangxi Province 330006 China; 6 Department of Pathology, The Second Affiliated Hospital of Nanchang University, Nanchang, Jiangxi Province 330006 China; 7 Department of Gynecology and Obstetrics, The Second Affiliated Hospital of Nanchang University, Nanchang, Jiangxi Province 330006 China; 8 Department of General Surgery, Jiangxi Provincial Cancer Hospital, Nanchang, Jiangxi Province 330029 China; 9 Jiangxi Provincial Clinical Research Center for General Surgery Disease, Nanchang, Jiangxi Province 330006 China

**Keywords:** PSMD7, Notch1, Pancreatic cancer, SOX2, Proliferation

## Abstract

**Background:**

Ubiquitination is a critical post-translational modification which can be reversed with an enzyme family known as deubiquitinating enzymes (DUBs). It has been reported that dysregulation of deubiquitination leads to carcinogenesis. As a member of the DUBs family, proteasome 26 S subunit non-ATPase 7 (PSMD7) serves as an underlying tumour-promoting factor in multiple cancers. However, the clinical significance and biological functions of PSMD7 in pancreatic cancer (PC) remain unclear.

**Results:**

In this study, we first reported frequent overexpression of PSMD7 in PC tissues, and high levels of PSMD7 were markedly linked to shorter survival and a malignant phenotype in PC patients. An array of in vitro and in vivo gain/loss-of-function tests revealed that PSMD7 facilitates the progression of PC cells. Additionally, we found that PSMD7 promotes PC cell progression by activating the Notch homolog 1 (Notch1) signalling. Interestingly, in PC cells, the inhibitory effect of PSMD7 knockdown on cellular processes was comparable to that observed upon Notch1 knockdown. Mechanistically, PSMD7 deubiquitinated and stabilised sex determining region Y (SRY)-box 2 (SOX2), a key mediator of Notch1 signalling. The stabilisation of SOX2, mediated by PSMD7, dramatically increased SOX2 protein levels, subsequently activating the Notch1 pathway. Finally, restoration of SOX2 expression abrogated the PSMD7-silenced antitumour effect.

**Conclusions:**

Taken together, our work identifies and validates PSMD7 as a promoter of PC progression through augmentation of the Notch1 signalling pathway mediated by SOX2. This finding suggests that PSMD7 holds promise as a potential therapeutic target for the management of this refractory disease.

**Supplementary Information:**

The online version contains supplementary material available at 10.1186/s13578-024-01213-9.

## Background

Pancreatic cancer (PC) is one of the deadliest malignant tumours within the category of digestive system tumors and poses a serious threat to human health. It is estimated that by 2025, PC will be the 3rd most frequent cause of cancer-related deaths [1]. The prognosis of patients with PC is poor, with an overall five-year survival rate of < 6% [2]. Although surgical resection remains the primary therapeutic approach for PC, a significant proportion of patients are precluded from undergoing this intervention due to late-stage diagnosis [3–5]. Revealing the critical mechanisms underlying PC growth and progression is imperative for identifying effective biomarkers for tumor diagnosis and devising innovative treatments to optimize prognosis.

Deubiquitination, as a post-translational modification, exerts regulatory effects on target protein activity, cellular fate, and intracellular signaling pathways [6]. Deubiquitination, the reverse process of ubiquitination, is mediated by a group of proteins known as deubiquitinating enzymes (DUBs) [7, 8]. DUBs exert their counteractive effect on ubiquitination by enzymatically cleaving monoubiquitin or polyubiquitin moieties from target proteins and serve critical functions in a variety of physiological processes [6]. Proteasome 26 S subunit non-ATPase 7 (PSMD7), also referred to as Mov34 or Rpn8, functions as an ATP-independent constituent of the 19 S regulatory subunit that interacts with PSMD14 to create a heterodimer, a functional complex in the proteasome that is essential for the degradation of ubiquitinated substrates. PSMD7 primarily acts in cell proliferation and apoptosis, as well as facilitating the nuclear export of various crucial proteins to the cytoplasm for protein stabilization [9, 10]. There is growing evidence that dysregulation of PSMD7 not only inactivates tumour suppressors, but also upregulates oncogene function in various cancers, leading to carcinogenesis [11–13]. In addition, mounting evidence suggests a robust association between elevated PSMD7 expression and enhanced cancer cell proliferation across various malignancies and can result in poor prognosis [14]. However, the mechanism by which PSMD7 facilitates PC cell proliferation remains to be examined.

Sex determining region Y (SRY)-box 2 (SOX2) is a highly conserved transcriptional regulator that serves a pivotal action in the maintenance of self-renewal and cellular totipotency of embryonic stem cells [15, 16]. Research has demonstrated that SOX2 is expressed in cells of various origins, including T cells, smooth muscle cells, and osteoblasts [17, 18]. In recent years, a growing body of investigations has consistently demonstrated that SOX2 plays a pivotal role in driving distant metastasis, tumour invasion, radioresistance, and drug resistance through the induction of epithelial-mesenchymal transition (EMT) in tumour epithelial cells [19]. Additionally, SOX2 is abnormally expressed in diverse types of tumours, including colorectal, gastric and pancreatic cancers [17, 20, 21]. Consequently, unravelling the SOX2 regulatory mechanism will give new insights into PC pathogenesis and novel strategies for the treatment of this lethal cancer.

Therefore, the objective of this study was to elucidate the pivotal roles played by SOX2 and PSMD7 in PC development and investigate the progression of this disease through the PSMD7/SOX2 /Notch1 axis.

## Methods

### Bioinformatics analysis

Gene Expression Profiling Interactive Analysis 2 (GEPIA2; http://gepia2.cancer-pku.cn/) was used to evaluate the differential expression of PSMD7 between normal and PC tissues. Using the Kaplan–Meier Plotter (http://kmplot.com/analysis/), Kaplan–Meier analysis of PSMD7 in PC was performed. A total of 179 PC samples with RNA-seq data (level 3), together with the corresponding clinical information, were derived from the TCGA database (https://portal.gdc.com).

### Patients and tumour specimens

PC samples were collected from 104 patients who *were* collected from undergoing resection surgery, and did not receive any chemotherapy before surgery at the Department of General Surgery, the Second Affiliated Hospital of Nanchang University, China. The trials were performed with each participant’s informed and written consent, and the research methodology was in line with the standards set forth in the Declaration of Helsinki. This study was approved by the Ethics Committee of the Second Affiliated Hospital of Nanchang University, China.

### Cell culture

The HPDE6-C7 (human normal pancreatic ductal epithelial) cell and five human PC cell lines (BxPC-3, AsPC-1, SW 1990, CFPAC-1, and PANC-1) were obtained from the Shanghai Institute of Cells of the Chinese Academy of Sciences. These cells were characterised using a cell bank of short tandem repeats. Cells were conventionally cultivated in DMEM medium with 10% foetal bovine serum (FBS, Gibco) at 5% CO_2_ and 37 °C.

### Cell transfection

The shPSMD7 expression and PSMD7 overexpression plasmids, as well as the shSOX2-expression and SOX2-overexpression plasmids, were designed and produced by GenePharma (Shanghai, China). Lipofectamine 3000 (Invitrogen, Carlsbad, CA, USA) was used for cell transfection. Reagents and plasmids used are listed in Supplementary Table 1.

### Knockdown of PSMD7 by RNA interference delivered by lentivirus

A negative control interfering sequence (TTCTCCGAACGTGTCACGT) and two short-hairpin RNA (shRNA) sequences (Supplementary Table 1) were designed and constructed by Focus Bioscience Co., Ltd (Shanghai, China). A pLKO-puro vector with a short hairpin RNA (shRNA) sequence was established. The lentivirus was packaged with Lipofectamine 3000 (Invitrogen, Carlsbad, CA, USA) and pLKO-pur plasmids and subsequently used to infect PANC-1 cells. Lentiviral supernatants were collected after 48 h, and PANC-1 cells were infected with polybrene (Sigma-Aldrich, USA) and selected with puromycin (Thermo Scientific, USA).

**Table 1:** The relationship between PSMD7 expression and clinicopathological characteristics in PC patients

Characteristics	No. of patients	PSMD7 Expression		p-value
		Low *n*=42	High *n*=62	
Age(years)				0.606
<60	39	17	22	
≥60	65	25	40	
Gender				0.339
Male	56	25	31	
Female	48	17	31	
Tumor size (cm)				**0.025**
≤3	58	29	29	
>3	46	13	33	
Lymph node metastasis				0.147
Negative	48	23	25	
Positive	56	19	37	
TNM stage				**0.038**
I and II	78	36	42	
III and IV	26	6	20	
Perineural invasion				0.056
Negative	29	16	13	
Positive	75	26	49	

Bold values are statistically significant, *p* < 0.05

### Quantitative real-time PCR

Extraction of RNA from tissues or cultured cells was carried out using RNAiso Plus (Takara, Japan), and the RNA was reverse transcribed to cDNA and amplified by PCR. The outcomes were evaluated using the 2^−△△Ct^ approach. All primers used are listed in Supplementary Table 1.

### Western blotting

Western blotting was performed as mentioned earlier [22]. The following antibodies were used in this study: rabbit SOX2 (1:5,00, ab171380, Abcam), rabbit Notch1 (1:1,000, 20687-1-AP, Proteintech), mouse HES1 (1:5,00, ab119776, Abcam), mouse GAPDH (1:1,000, 60004-1-Ig, Proteintech), and rabbit PSMD7 (1:1,000, 16034-1-AP, Proteintech).

### Immunoprecipitation

The immunoprecipitation assay was performed as described by Yuan et al. [23]. Cells were lysed using NP-40 lysis buffer. Western blotting (input) was performed to analyse the protein lysates (5%). The residual lysate was later added to protein A/G agarose beads that were pre-coupled with the antibody and cultivated under a temperature of 4 °C for 4 h. The beads were rinsed and boiled in 2 × SDS upwelling buffer for western blotting.

### Immunohistochemistry analysis

Immunohistochemistry (IHC) analyses of SOX2, Ki-67, and PSMD7 were performed as mentioned previously [24]. The following antibodies were used: rabbit anti-Ki-67 (1:2,00, 28074-1-AP, Proteintech), rabbit Panti-SMD7 (1:2,00, ab140428, Abcam) and rabbit anti-SOX2 (1:2,00, ab97959, Abcam).

### Cell counting Kit-8 (CCK-8) assay

The cells after 48 h of transfection were collected and plated into 96-well plates at 2000 cells per well. After cultured for 24 h, 48 h, 72 h, and 96 h, respectively, 10 µL of CCK-8 solution (Dojindo Molecular Technologies, Kumamoto, Japan) was added per well for incubation for 2 h, and then, the optical density (OD) value of each well was measured in the microplate reader at 450 nm absorption wavelength.

### 5-Ethynyl-2’-deoxyuridine (EdU) assay

The cells were incubated with EdU (Ribobio, Guangzhou, China) for 2 h. The cells were treated with 100 µL of 1× Apollo reaction cocktail for half an hour after three PBS rinses. The cells in each well were stained for 30 min with 100 µL of 1× Hoechst 33342 to determine DNA content. The cells were then observed under a fluorescence microscope.

### In vivo tumor growth assay

We obtained 4–6-week-old nude mice from the Institute of Model Zoology, Nanjing University. All assays were performed according to the National Institutes of Health (NIH) guidelines for humane care and utilisation of laboratory animals and were authorised by the Institutional Animal Care and Use Committee at Nanchang University. The cell concentration was adjusted to cells (1 × 10^6^). After sterilising the skin into both the legs of the nude mice with 75% alcohol, both shNC (Vector) cells and shPSMD7 (Flag-PSMD7) cells were injected into the same mouse but on different leg area of the nude mice. Nude mice were regularly observed for tumour growth and general health, and the weight and volume of the tumours were recorded.

### Co-IP and ubiquitination test in vivo

In the Co-IP assay, cell lysates were cultured overnight under a temperature of 4 °C with given primary antibodies and subsequently incubated with protein A/G sepharose beads (Santa Cruz, USA). The coprecipitated proteins were then collected and recognised by immunoblotting with the given antibodies. For the ubiquitination assay in vivo, cells were co-transfected with the above plasmids. The treatment of cells following transfection was conducted with proteasome inhibitor (MG132, 15 µM) for 12 h. Finally, the cells were lysed using a protocol identical to that used for the Co-IP assay, and immunoprecipitation and western blotting were performed.

### Proteasome activity assay

The Proteasomal activity was measured using a proteasome activity assay kit (Abcam; ab107921). PANC-1and SW 1990 cells were transfected with indicated plasmids for 48 h and shRNA for 72 h, followed by trypsin digestion and quantification. The cells were subsequently lysed in 90 µL lysis buffer (0.5% NP-40 in PBS), and the lysates were centrifuged at 16,000 × g for 15 min at 4 °C, then, the supernatants were collected in new vials. The proteasomal activity of the supernatants was determined by assaying the cleavage of a fluorogenic peptide substrate Suc-LLVY-AMC according to the manufacturer’s instruction. The substrate peptides were incubated with the cell lysates at 37 °C for 1 h, and the fluorescence intensity was measured at the end of the assay using a BioTek microplate reader (excitation/emission = 350/440 nm).

### LC-MS/MS analysis

The same co-IP method was used to extract anti-PSMD7 immunoprecipitates from PANC-1 cell lysates. Electrophoresis was conducted with electrophoresis sample buffer (2 × 20 µL). Subsequently, SDS-PAGE gels were stained with Coomassie Brilliant Blue for 3 h and cleaned with an eluent overnight. The stained gel was used for LC-MS/MS analysis as previously described [23].

### Dual-luciferase reporter assay

For the luciferase reporter assay, pGL3.0-basic-Notch1 reporter plasmids and the internal control plasmid pGL3.0-basic-vector were transfected into PC cells grown to 70% confluence in 24-well plates. The Notch1 promotors sequences are as follows: Notch1-F: 5’-GCTAGCCCGGGCTCGAGACCGAATGGGCCACTCTCTTC-3’, Notch1-R: 5’-ACCGGAATGCCAAGCTTCACTAGTGAGGCTCAGAGTCGAG-3’, The PSMD7 expression plasmid or empty vector were co-transfected for 48 h, and reporter gene activity was assayed using the Dual Luciferase Assay System (Promega; E1910) according to the manufacturer’s instructions. The activity of the pGL3.0 with the Notch1 promoter-luciferase reporter normalized to that of the pGL3.0-basic-Luc reporter was compared between PC cells transfected with PSMD7 expression plasmid or empty vector. Using a dual-luciferase reporter assay system (Promega, Madison, WI, USA), we determined luciferase activity, which was standardised to Renilla luciferase activity. The experiment was repeated three times.

### *Statistical analysis*

SPSS 22.0 software was employed for statistical processing of data, and all results acquired were denoted as standard deviation (SD). Comparisons between two and multiple groups were performed using Student’s t-test, along with ANOVA and post-hoc tests. Correlation was analysed using Spearman’s correlation analysis, with *p* < 0.05 as the critical value for statistical significance.

## Results

### High expression of PSMD7 is associated with poor PC prognosis

**Fig. 1:**
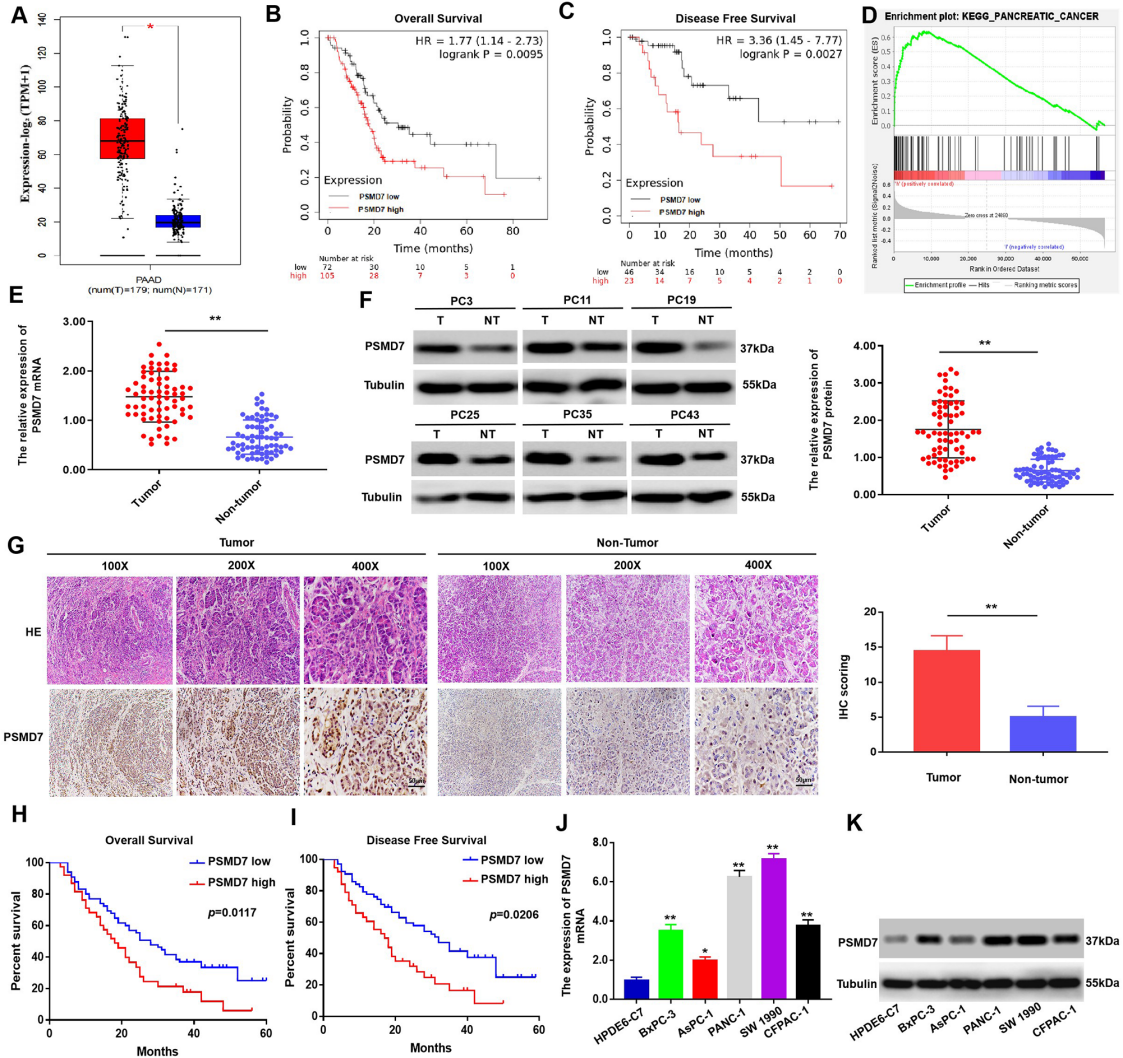
High expression of PSMD7 is associated with poor prognosis in PC. (**A**) Expression of PSMD7 in pancreatic non-tumour tissues (*n* = 171) and PC patient specimens (*n* = 179) evaluated via the GEPIA webtool. (**B)** and **(C)** Overall survival ( *p* = 0.0095) and disease-free survival ( *p* = 0.0027) times of patients with low versus high expression of PSMD7 in PC. (**D)** GSEA outcomes were graphed to show the association between poorly regulated genes and PSMD7 expression in PC. (**E)** and **(F)** Western blotting and qRT-PCR assay of PSMD7 expression in human PC tissues (*n* = 70), together with neighbouring normal tissues (*n* = 70). **G**, Quantification and representative immunohistochemistry (IHC) images of PSMD7 expression in human PC tissues, together with neighbouring normal tissues. (**H)** and **(I)** Kaplan–Meier analysis of the overall survival ( *p* = 0.0117) and disease-free survival ( *p* = 0.0206) time of PC patients with various PSMD7 expression. (**J)** and **(K)** The PSMD7 expression in BxPC-3, AsPC-1, HPDEC, CFPAC-1, PANC-1, and SW 1990 cells analysed via qRT-PCR and western blotting. *p < 0.05, **p < 0.01

To investigate the implications of PSMD7 in PC, we first assayed PSMD7 expression. Data obtained from Gene Expression Profiling Interactive Analysis (GEPIA) (http://gepia.cancer-pku.cn/) revealed that the expression of PSMD7 was higher in PC tissues (T) than in non-tumour tissues (NT) (Fig. 1A and Supplementary Fig. 1A). Remarkably, PSMD7 expression was significantly associated with both overall survival (*p* = 0.0095, Fig. 1B) and disease-free rates (*p* = 0.0027, Fig. 1C). GSEA also indicated that the dysregulation of gene expression in PC was linked to PSMD7 (Fig. 1D). Furthermore, ROC curve analysis showed that PSMD7 had high sensitivity and specificity in the diagnosis of PC (AUC = 0.9742, 95% CI: 0.956–0.991, *p* < 0.01) (Supplementary Fig. 1B). Subsequently, we analyzed 70 PC specimens newly collected in recent years by qRT-PCR and Western blotting (Fig. 1E, F). The results showed that the expression of PSMD7 protein and mRNA in PC tissues was higher than that in adjacent tissues. For further evaluation of the association of PSMD7 expression with the clinicopathologic characteristics of PC, IHC assay was conducted on 104 human PC specimens to examine PSMD7 expression (Fig. 1G). The IHC results showed that the expression of PSMD7 in PC tissues was significantly higher than that in adjacent tissues.

Clinicopathological correlation analysis revealed a correlation between increased PSMD7 expression, TNM stage, and tumour size (Table 1, Supplyment Table 2). Kaplan-Meier analysis revealed a significant correlation between elevated PSMD7 levels and reduced overall survival as well as disease-free survival (Fig. 1H, I). Furthermore, PSMD7 was highly expressed in five PC cell lines (SW 1990, PANC-1, CFPAC-1, AsPC-1, and BxPC-3) compared to in normal pancreatic cells (HPDE6-C7) (Fig. 1J, K). These findings demonstrate that PSMD7 is commonly elevated in PC and is significantly associated with an unfavorable prognosis in individuals afflicted with this disease.

### PSMD7 facilitates PC cell growth in vitro and in vivo

**Fig. 2:**
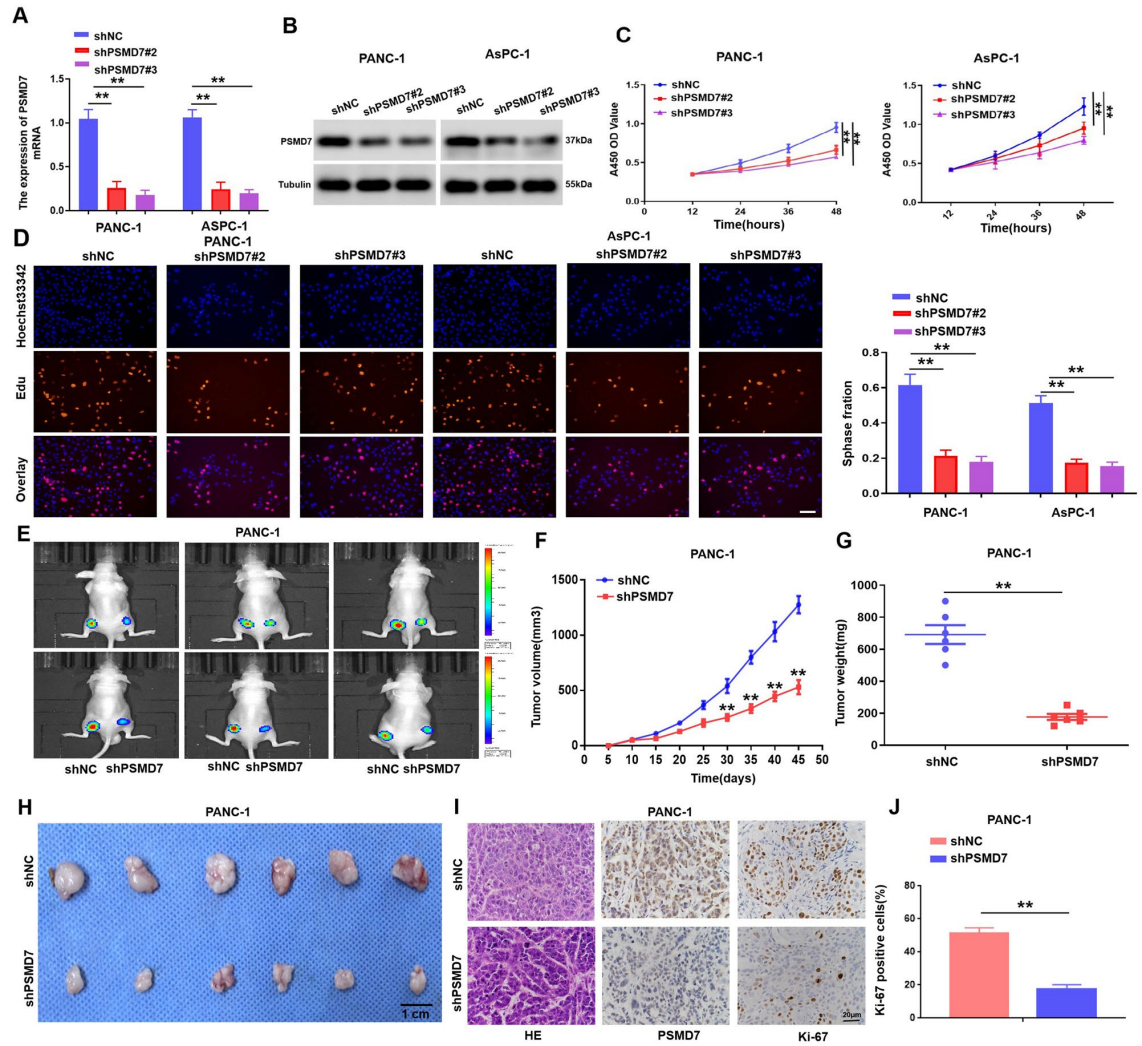
Kowndown of PSMD7 expression restrains the PC cell proliferative capacity in vitro and in vivo. (**A)** The mRNA of PSMD7 in AsPC-1 and PANC-1 cells transfected with shPSMD7 or shNC assayed via qRT-PCR. (**B)** PSMD7 knockdown in AsPC-1 and PANC-1 cells was validated via western blotting. (**C)** The proliferative abilities of AsPC-1 and PANC-1 PC cells with shNC or shPSMD7 treatment was detected separately via CCK-8. (**D)** Proliferative capacities of AsPC-1 and PANC-1 cells that were transfected with shPSMD7 or shNC were assayed with EdU, and the scale bar represents 50 μm, **p < 0.01. (**E)** Evaluation of mice that were injected with luciferase-expressing PANC-1/shPSMD7 or PANC-1/shNC cells via the IVIS imaging system. (**F)** Tumour volumes of the PANC-1/shPSMD7 and PANC-1/shNC groups. Volumes of tumours are expressed as the mean ± SD, *n* = 6. (**G)** Tumour weight of the shPSMD7 and shNC groups. *n* = 6. **(H)** The images of tumors the PANC-1/shPSMD7 and PANC-1/shNC groups. (**I)** and (**J)** Immunohistochemistry (IHC) analysis of PSMD7 and Ki-67 expression in tumours of the PANC-1/shPSMD7 and PANC-1/shNC groups, scale bar denotes 100 μm. *n* = 3, *p < 0.05, **p < 0.01

**Fig. 3:**
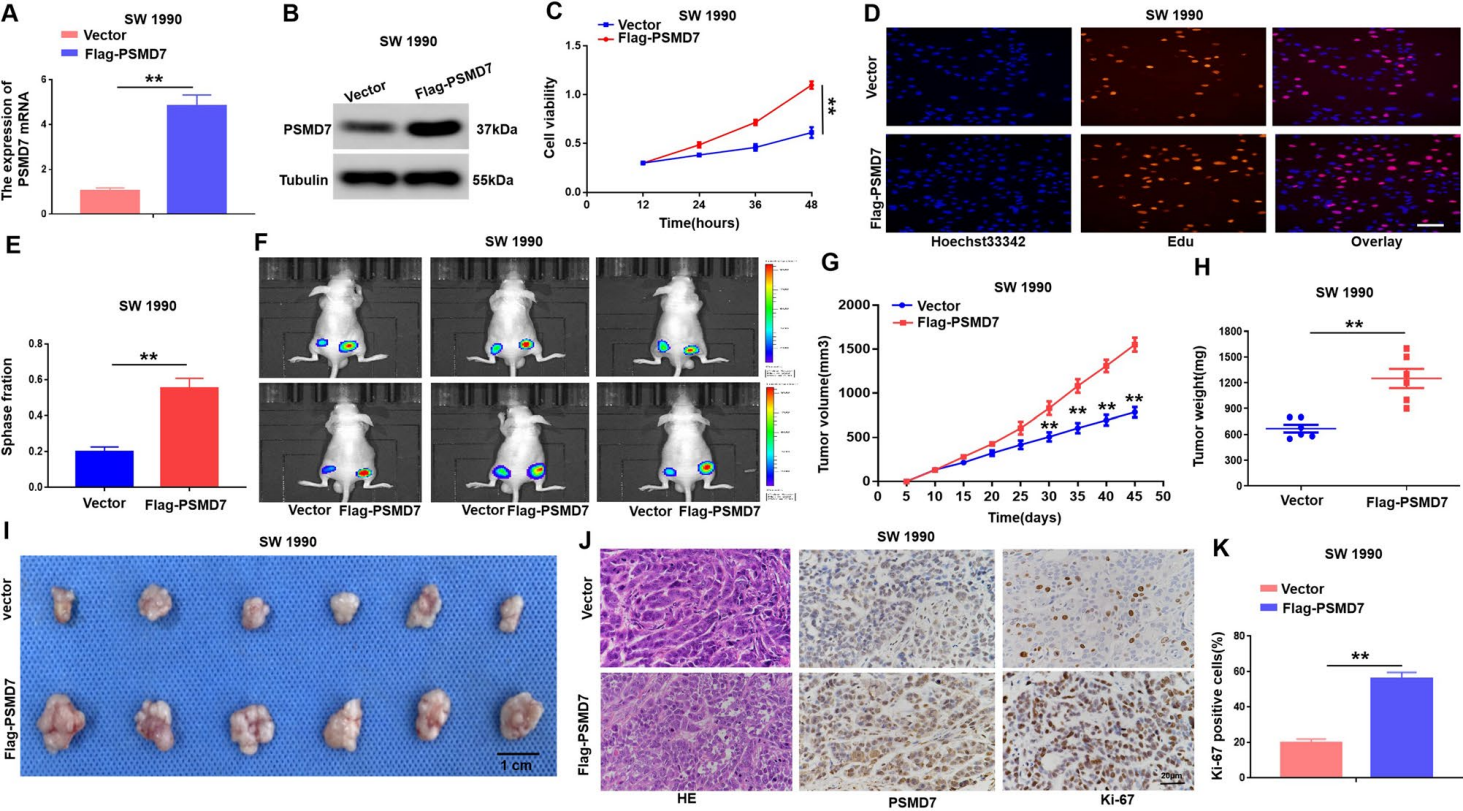
Overexpression of PSMD7 promotes PC proliferation. **(A)** and **(B)** PSMD7 mRNA along with protein levels in SW 1990 cells transfected with Vector or Flag-PSMD7 plasmids were detected by qRT-PCR as well as western blotting. Tubulin was employed as the loading control. (**C)** CCK-8 assay was employed to examine the difference in cell viability between both groups. (**D)** and **(E)** EdU staining was utilized to establish the impact of PSMD7 overexpression on the PC cell proliferation rate. Scale bar denotes 50 μm, **p < 0.01. (**F)** Mice injected with luciferase-expressing SW 1990/Flag-PSMD7 or SW 1990/Vector cells were investigated through IVIS imaging system. (**G)** Tumour volumes for SW 1990/Flag-PSMD7 or SW 1990/Vector groups. Volumes of tumours are expressed as mean ± SD, *n* = 6, **p < 0.01. (**H)** Tumour weights in the Flag-PSMD7 and Vector groups, *n* = 6, **p < 0.01. (**I)** Tumour volumes for SW 1990/Flag-PSMD7 or SW 1990/Vector groups. (**J)** and **(K)**, immunohistochemistry (IHC) analysis of Ki-67 and PSMD7 expression in tumours of the SW 1990/Flag-PSMD7 or SW 1990/Vector group, scale bar is 20 μm. *n* = 3, *p < 0.05, **p < 0.01

To understand the impact of increased PSMD7 expression on PC carcinogenesis, we transfected shPSMD7#1, shPSMD7#2,shPSMD7#3 and shNC interference plasmids into PANC-1 cells, and the qRT-PCR and Western blotting results showed that shPSMD7#2 and shPSMD7#3 interference plasmids had obvious effect on knockout (Supplymentment Fig. 1C, D). Then, a in-of-function test was performed by decreasing PSMD7 expression in the AsPC-1 and PANC-1 cell lines and upregulating PSMD7 expression in the SW 1990 cell line (Figs. 2A and B and 3A and B ). The role of PSMD7 in PC cell proliferation was investigated using EdU and CCK-8 assays. As presented in Figs. 2C and D and 3C-E, in PC cells with PSMD7 knockdown, the cell proliferation capacity was obviously lowered, whereas in PC cells with high PSMD7 expression, the cell proliferation capacity was obviously enhanced. These findings suggest that PSMD7 plays a pivotal role in facilitating PC cell proliferation in vitro.

To investigate the impact of PSMD7 on in vivo tumor growth, we injected PANC-1 and SW 1990 cells stably transfected with PSMD7-interfering lentivirus or PSMD7-overexpressing lentivirus into the subcutis of both legs of nude mice, respectively. The results of in vivo experiments demonstrated that the suppression of PSMD7 led to a significant inhibition of tumour growth (Fig. 2E). Moreover, the PSMD7-knockout group exhibited significantly reduced tumour weight and volume compared to the control group (Fig. 2F, G and H). IHC analysis demonstrated that the knockdown of PSMD7 resulted in a reduction in the number of Ki-67-positive cells within the tumor (Fig. 2I, J). However, upregulation of PSMD7 increased tumour growth (Fig. 3F-K). The data presented herein provide compelling evidence for the pivotal oncogenic role of PSMD7 in promoting the growth of PC cells.

### Notch1 pathway as a PSMD7 downstream component facilitates the role of PSMD7 in PC cells

**Fig. 4:**
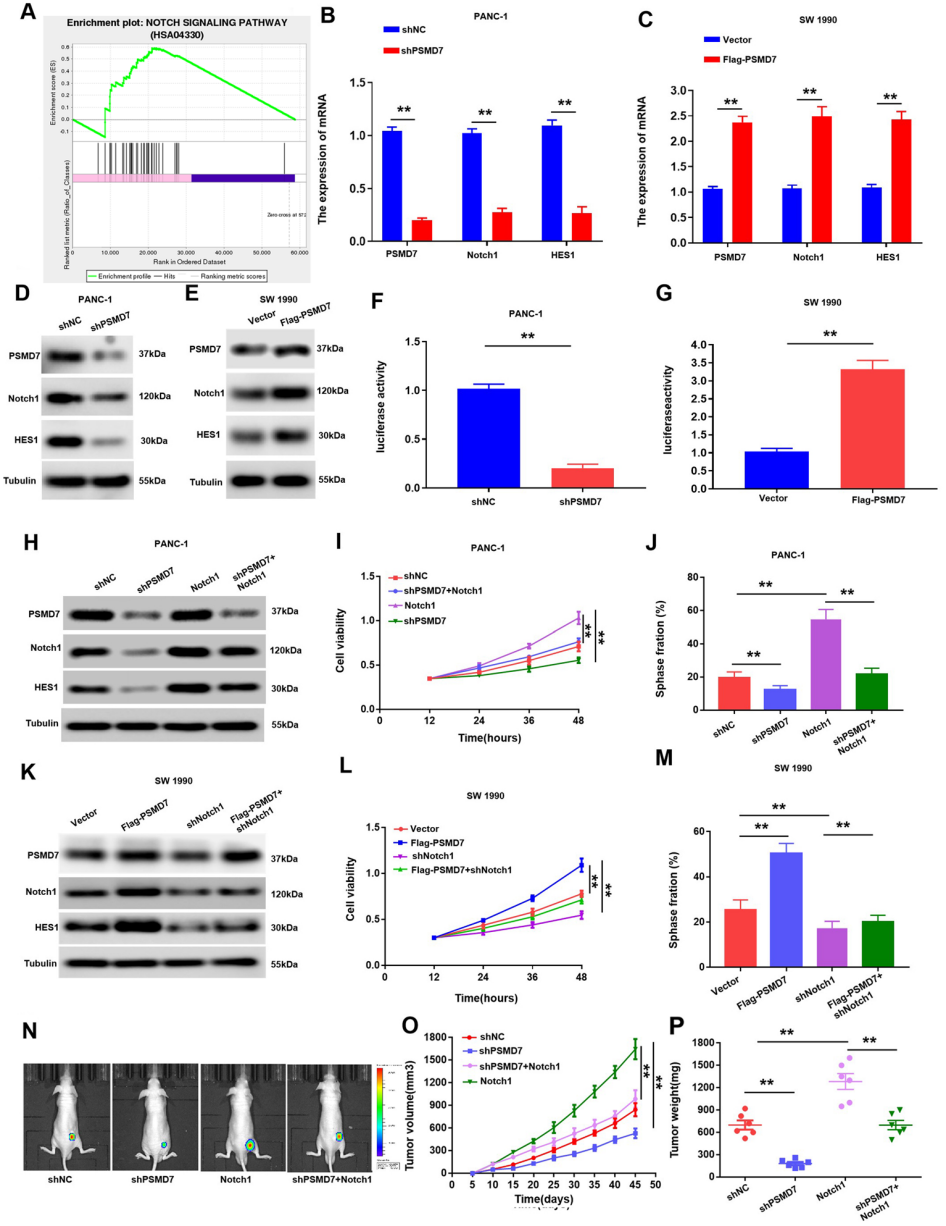
Notch1 pathway, downstream component of PSMD7, facilitates the role of PSMD7 in PC cells. (**A)** GSEA outcomes were graphed to show the association between genes linked to the Notch1 signalling pathway and PSMD7 expression. (**B)** and **(C)** The HES1 and Notch1 mRNA expression levels were assayed by qRT-PCR with PSMD7 overexpression in SW 1990 cells or knockdown in PANC-1 cells. (**D)** and **(E)** Protein levels of Notch1 along with its target genes (including HES1) were measured via western blotting after PSMD7 overexpression in SW 1990 cells or silencing in PANC-1 cells. (**F)** and **(G)** Luciferase activities were examined after transfection of sh-PSMD7 and the corresponding luciferase reporter plasmids in PSMD7-overexpressing SW 1990 cells or PANC-1 cells. (**H)** Expression of HES1, Notch1, and PSMD7 in PC cells that were co-transfected with Notch1 and sh-PSMD7. (**I)** and **(J)** EdU and CCK-8 analysis of cell proliferation in PANC-1 cells that expressed shPSMD7 or shNC, with or without overexpression of Notch1. (**K)** Expression of HES1, Notch1, and PSMD7 in PC cells that were co-transfected with sh-Notch1 and Flag-PSMD7. (**L)** and **(M)** EdU and CCK-8 analysis of cell proliferation in SW 1990 cells that expressed exogenous PSMD7 or control vector, with or without shNotch1 transfection. (**N)** Mice that were injected with luciferase-expressing PANC-1/Notch1, PANC-1/shPSMD7, PANC-1/shNC, or PANC-1/shPSMD7 + Notch1 cells were evaluated via IVIS imaging system. (**O)** Tumour volumes in the PANC-1/Notch1, PANC-1/shPSMD7, PANC-1/shNC, or PANC-1/shPSMD7 + Notch1 groups. Volumes of tumours are represented as mean ± SD, *n* = 6; (**P)** Tumour weight in the PANC-1/Notch1, PANC-1/shPSMD7, PANC-1/shNC, or PANC-1/shPSMD7 + Notch1 group. *n* = 6. *p < 0.05, **p < 0.01

To detect the latent molecular mechanisms of PSMD7 action, we first carried out GSEA in the TCGA database to investigate potential associations between PSMD7 and a variety of signalling pathways. As shown in Fig. 4A, in PC samples with high levels of PSMD7, the gene set Hallmark_Notch1_Targets was markedly enriched, indicating a strong association between the Notch1 pathway and elevated levels of PSMD7 in PC. We assumed that the Notch1 signalling pathway was activated by PSMD7 because the former had the highest enrichment score. To verify this hypothesis, western blotting and qRT-PCR analyses were conducted, and silencing PSMD7 was found to repress the protein and mRNA levels of the Notch1 target gene HES1 (Fig. 4B, D). Conversely, overexpression of PSMD7 resulted in enhanced protein and mRNA levels of Notch1, together with its target gene, HES1 (Fig. 4C, E). To further validate the link between NOTCH1 and PSMD7 signaling, dual luciferase assays in SW 1990 and PANC-1 cells showed that the transcriptional activity of NOTCH1 was repressed by PSMD7 silencing, whereas that of NOTCH1 was activated by PSMD7 up-regulation (Fig. 4F, G). Collectively, these data demonstrate that PSMD7 may function by augmenting the Notch1 signalling pathway.

To investigate the involvement of the Notch1 signaling pathway in the oncogenic role of PSMD7 in PC, the plasmid for Notch1 overexpression was first transfected into PSMD7 knockdown cells, and subsequently, HES1, Notch1, and PSMD7 protein expression was analysed by western blotting. The proliferative capacity of the cells was evaluated using EdU and CCK-8 assays. Our data demonstrated that PSMD7 downregulation suppressed HES1 and Notch1 expression, whereas Notch1 up-regulation attenuated the decrease in HES1 and Notch1 expression resulting from the knockdown of PSMD7 (Fig. 4H). CCK-8 and EdU assays revealed that knockdown of PSMD7 repressed proliferation, whereas overexpression of the Notch1 gene alleviated the reduction in cell proliferation resulting from PSMD7 knockdown (Fig. 4I, J). Conversely, PSMD7 overexpression markedly enhanced Notch1 expression, whereas knockdown of Notch1 remarkably inhibited this increase in PSMD7-induced Notch1 expression (Fig. 4K). Additionally, knockdown of Notch1 attenuated cell proliferation resulting from overexpression of PSMD7 (Fig. 4L, M). These results revealed that Notch1 is a pivotal signalling pathway for the proliferation of PC cells induced by PSMD7. Furthermore, in vivo investigations demonstrated that rescuing the expression of Notch1 attenuated the reduction in tumour growth induced by PSMD7 knockdown (Fig. 4N-P). In conclusion, our findings revealed that Notch1 is regulated by PSMD7 and is a crucial mediator of PC cell growth evoked by PSMD7.

### PSMD7 activates Notch1 pathway by modulating SOX2 expression

**Fig. 5:**
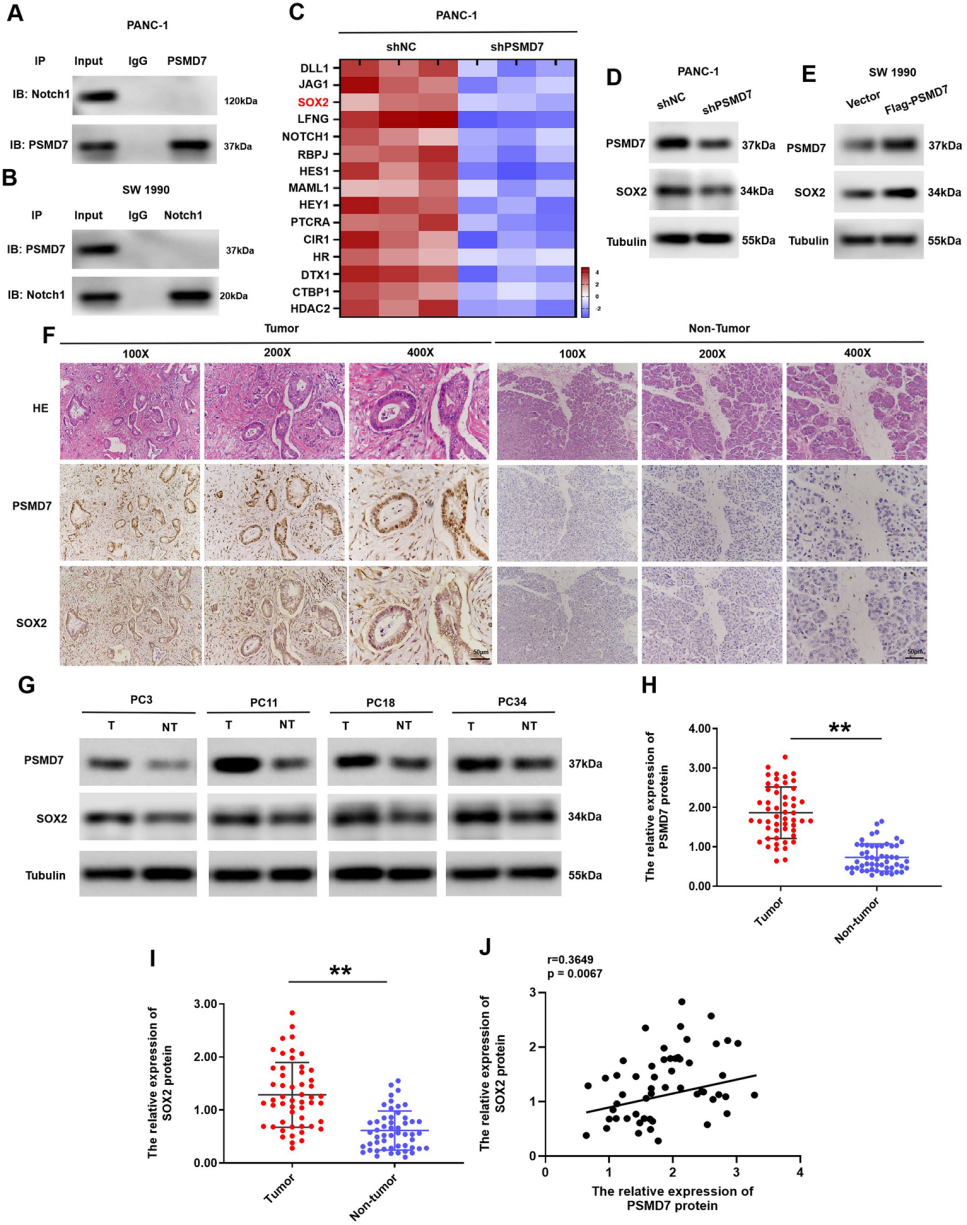
PSMD7 activates Notch1 pathway by modulating SOX2 expression

It has been reported that PSMD7 acts by interacting with various substrates. To further elucidate the mechanism of PSMD7 modulation of Notch1 in PC cells, we initially investigated the potential direct interaction between Notch1 and PSMD7. However, co-immunoprecipitation (co-IP) analysis did not reveal any direct interaction (Fig. 5A, B). Subsequently, to identify the intrinsic mechanism of PSMD7 activation of the Notch1 signalling pathway in PC, large-scale proteomic assays were carried out in PSMD7-silenced PC cells using TMT-based LC-MS/MS analysis. SOX2 protein expression was reduced in PSMD7-silenced PC cells (Fig. 5C). Notably, SOX2 has been reported to be an essential transcription factor for the positive transcriptional regulation of Notch1 [24]. To investigated SOX2 regulates the NOTCH1 signal in PC cells, we used SOX2 knockdown PANC-1 cell lines and found that SOX2 downregulation decreased the mRNA levels of Notch1 and downstream targets Hes1. On the contrary, SOX2 upregulation enhanced the mRNA levels of Notch1 and downstream targets Hes1 (Supplementary Fig. 2A and B). Furthermore, dual-luciferase detection in SW 1990 and PANC-1 cells demonstrated that NOTCH1 transcriptional activity was inhibited by SOX2 silencing, whereas NOTCH1 transcriptional activity was repressed by SOX2 upregulation (Supplementary Fig. 2C and D). These findings led us to speculate that SOX2 could activate Notch1 signalling. As expected, SOX2 protein expression was markedly reduced in PSMD7-silenced PC cells (Fig. 5D). In comparison, the expression of the SOX2 protein increased in PSMD7-overexpressing PC cells, implying that SOX2 activated the Notch1 signalling pathway mediated by PSMD7 (Fig. 5E). To validate the modulation of SOX2 mediated by PSMD7 in clinical PC specimens, SOX2 expression in PC tissues was examined by IHC. IHC staining showed that SOX2 accumulated in PC tissues with high PSMD7 expression (Fig. 5F). To further corroborate the connection between SOX2 and PSMD7, we tested 52 PC specimens newly collected in recent years using western blotting (Fig. 5G-I). Importantly, statistical analyses illustrated that SOX2 protein levels were positively associated with PSMD7 protein levels in PC tissues (Fig. 5J). Collectively, the findings collectively demonstrate that PSMD7 activates the Notch1 pathway by modulating the expression of SOX2.

**(A)** and **(B)** Co-IP test was implemented to identify the protein binding of Notch1 and PSMD7 in SW 1990 and PANC-1 cells, separately. (**C)** Heatmap displaying the first 15 most differentially expressed genes in the PANC-1 cells that were transfected with shPSMD7 or shNC. (**D)** and **(E)** Protein expression of PSMD7 and SOX2 in shNC or shPSMD7 in PANC-1 cells and exogenous PSMD7 or vector in SW 1990 cells examined via western blotting. (**F)** Representative plots of immunohistochemistry (IHC) staining for SOX2 and PSMD7 in clinical PC samples. 100× plots with a scale bar of 200 μm and 400× plots with a scale bar of 50 μm. (**G-I)** western blotting assay of PSMD7 expression in human PC tissues (*n* = 52), together with neighbouring normal tissues (*n* = 52). **p < 0.01. (**J)** Correlations of data from western blotting of PSMD7 expression with respect to the level of SOX2. *r* = 0.3649, *p* = 0.0067. *p < 0.05, **p < 0.01.

### PSMD7 interacts with SOX2

**Fig. 6:**
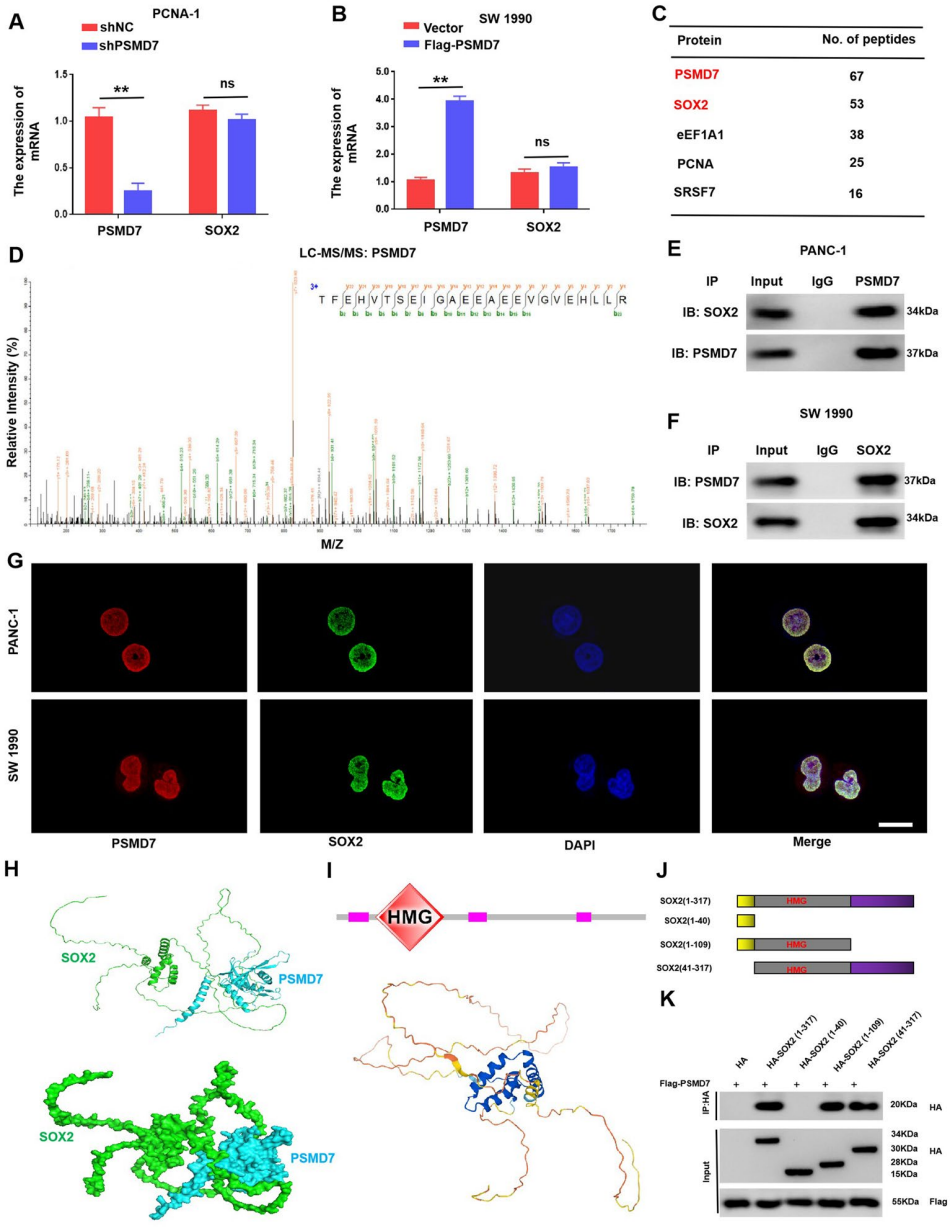
PSMD7 interacts with SOX2. (**A)** and **(B)** qRT-PCR analysis of PSMD7 and SOX2 mRNA expression in PANC-1 cells transfected with either shNC or shPSMD7, and SW 1990 cells transfected with exogenous PSMD7 or a control vector. *n* = 3; ns, not significant. **p < 0.01. (**C)** The first 5 proteins that co-precipitated with PSMD7 were identified via LC-MS/MS. #PSMs with matching peptide profiles. (**D)** Mass spectra displaying distinct peptides of PSMD7 characterized by 2D-LC-MS/MS after immunoprecipitation of PANC-1 cell lysates with anti-PSMD7. (**E)** and **(F)** Immunoprecipitation of SW 1990 and PANC-1 cell lysates with control IgG, anti-SOX2, or anti-PSMD7 antibodies. (**G)** SW 1990 and PANC-1 cells were fixed and stained using SOX2 (green) and PSMD7 (red) antibodies. Cell nuclei were stained using DAPI (blue). Scale bar: 20 μm. (**H)** Top ranked docking conformations and 3D structures of SOX2 and PSMD7. SOX2 and PSMD7 are displayed in cyan and green, respectively. (**I)** Schematic illustration of SOX2 structure. (**J)** Diagram of SOX2 truncated mutant constructs. (**K)** HMG region of SOX2 was required for interaction with PSMD7. *p < 0.05, **p < 0.01

To identify how PSMD7 modulates SOX2 expression, qRT-PCR was performed to investigate SOX2 mRNA expression in PSMD7-overexpressing and PSMD7-knockdown PC cells. The results revealed that PSMD7 did not affect SOX2 mRNA levels, indicating that PSMD7 may control SOX2 expression at the post-transcriptional level rather than at the transcriptional level (Fig. 6A, B). The protein interactome was further explored via Co-IP mass spectrometry, which revealed that PSMD7 binds to SOX2 (Fig. 6C, D). Western blotting and endogenous IP analyses were subsequently performed to verify that PSMD7 interacted with SOX2 (Fig. 6E, F). Additionally, the co-localization of SOX2 and PSMD7 in pancreatic cancer cells was confirmed by confocal microscopy, providing further substantiation for the protein-protein interaction (Fig. 6G). Docking analysis revealed binding as well as interactions between SOX2 and PSMD7 (Fig. 6H). An array of SOX2 truncated plasmids tagged with haemagglutinin (HA) markers was constructed to characterise the specific structural domains that interact directly with PSMD7. SOX2 mainly contains two domains: the HMG (high mobility group) domain at the N-terminal, which is a DNA binding domain, and the TAD (trans-activation domain) domain at the C-terminal. Based on these structural information, the truncated form of the SOX2 mutant is constructed to determine the domain of its interaction with PSMD7. The subsequent localization experiments showed that the HMG domain of SOX2, the nterminal region of SOX2 (amino acid 41–109), was related to PSMD7 (Fig. 6I-K). Hence, our data indicate that there is an interaction between PSMD7 and SOX2.

### PSMD7 stabilizes SOX2 protein expression via suppressing SOX2 degradation mediated by proteasome

**Fig. 7:**
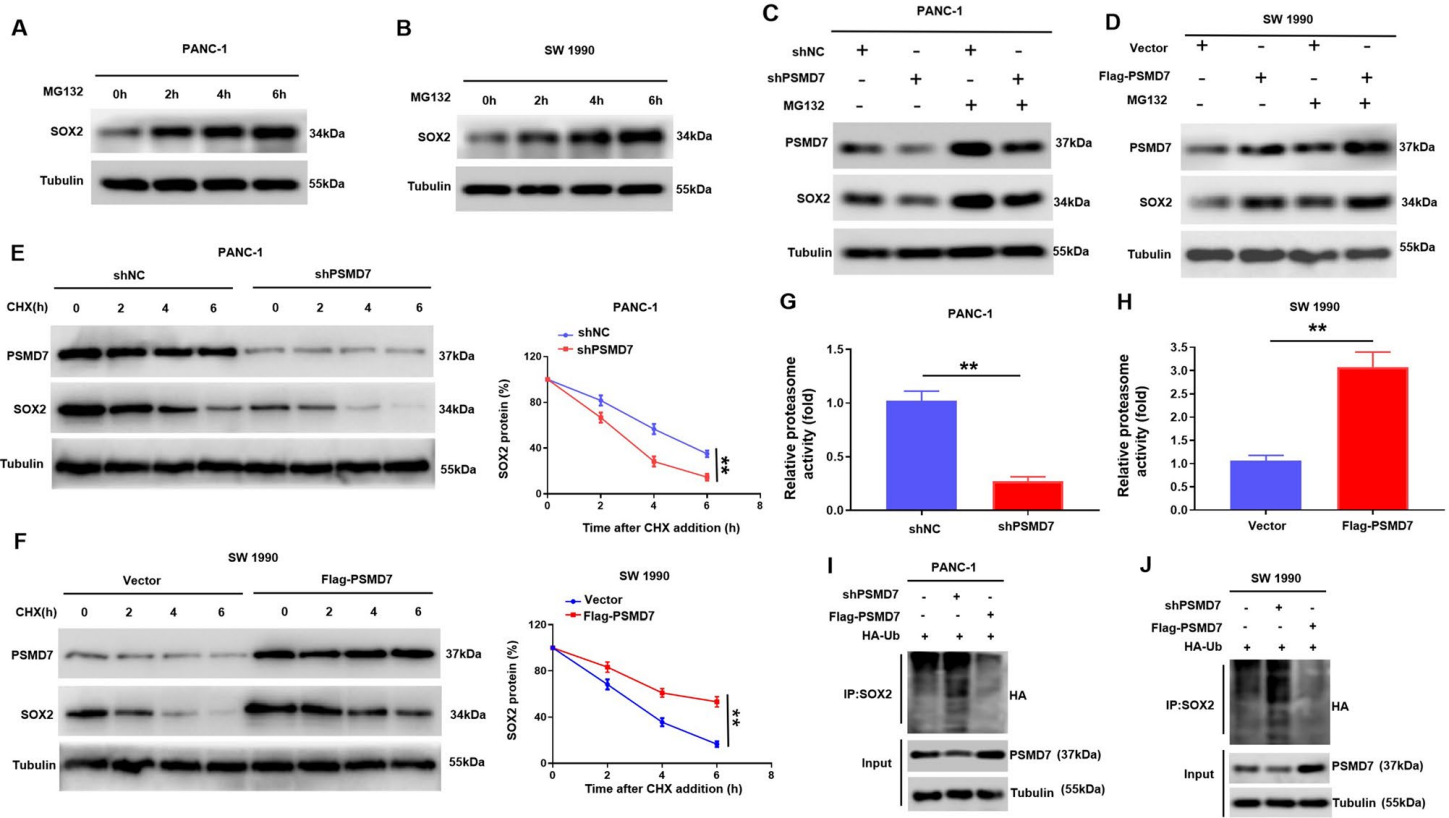
PSMD7 stabilizes SOX2 protein expression via suppressing SOX2 degradation mediated by proteasome. (**A**) and (**B**) SOX2 protein levels at various times were measured by western blotting after MG132 addition (10 µM) to SW 1990 and PANC-1 cells. (**C)** and **(D)** Western blot analysis of SOX2 and PSMD7 protein expression in PANC-1 cells transfected with shPSMD7 or shNC and SW 1990 cells transfected with exogenous PSMD7 or a control vector, with or without treatment with 10 µM MG132. (**E)** and (**F)** PANC-1 cells transfected with shPSMD7 or shNC and SW 1990 cells transfected with exogenous PSMD7 or a control vector were subjected to treatment with 20 µg/mL CHX, followed by assessment of SOX2 protein levels using western blotting. *n* = 3, **p < 0.01. (**G)** and **(H)** PANC-1 and SW 1990 cells were treated with shPSMD7 or PSMD7 for 72 h and the intracellular proteasome activity in the treated cells were assessed using proteasome activity fluorometric assay kit. Experiment was repeated three times. A statistically significant difference in the proteasome activity in cells treated with SAHA vs. without SAHA (control) is denoted by *p < 0.05, **p < 0.01. (**I)** and **(J)** Lysates of SW 1990 along with PANC-1 cells that were transfected with Flag-PSMD7, sh-PSMD7, and HA- ubiquitin (HA-Ub) were analysed via immunoblotting and then immunoprecipitated using anti-SOX2 and probed with anti-HA. *p < 0.05, **p < 0.01

Since PSMD7 is a deubiquitylase, we speculated that PSMD7 might modulate the degradation and ubiquitylation of SOX2 in PC. We found that MG132, a proteasome inhibitor, repressed the degradation of SOX2 (Fig. 7A, B). These findings imply that SOX2 undergoes proteasomal degradation in PC cells. Consequently, we examined whether PSMD7 modulates SOX2 protein stability by influencing proteasomal degradation. As presented in Fig. 7C, PSMD7 knockdown caused a pronounced decrease in SOX2, whereas treatment with MG132 completely blocked the decrease in SOX2 resulting from the knockdown of PSMD7. In contrast, treatment with MG132 inhibited the increase in SOX2 expression induced by the overexpression of PSMD7 (Fig. 7D). Additionally, the influence of PSMD7 on the half-life of SOX2 in CHX-treated PC cells was characterised. The findings suggest that the knockdown of PSMD7 shortened the half-life of the SOX2 protein, whereas the overexpression of PSMD7 prolonged its half-life (Fig. 7E, F). Furthermore, we conducted an in vitro proteasome activity assay and found that the knockdown of PSMD7 decreased the expression activity of proteasome in PANC-1 cells (Fig. 7G). In contrast, the overexpression of PSMD7 increased the expression activity of proteasome in SW 1990 cells (Fig. 7H). Co-IP analysis demonstrated that PSMD7 knockdown enhanced the endogenous SOX2 ubiquitination level, whereas overexpression of PSMD7 lowered it (Fig. 7I, J). Taken together, these findings indicate that PSMD7 represses SOX2 protein degradation, thereby contributing to SOX2 stability.

### PSMD7 oncogenesis relies on the SOX2-Notch1 pathway

**Fig. 8:**
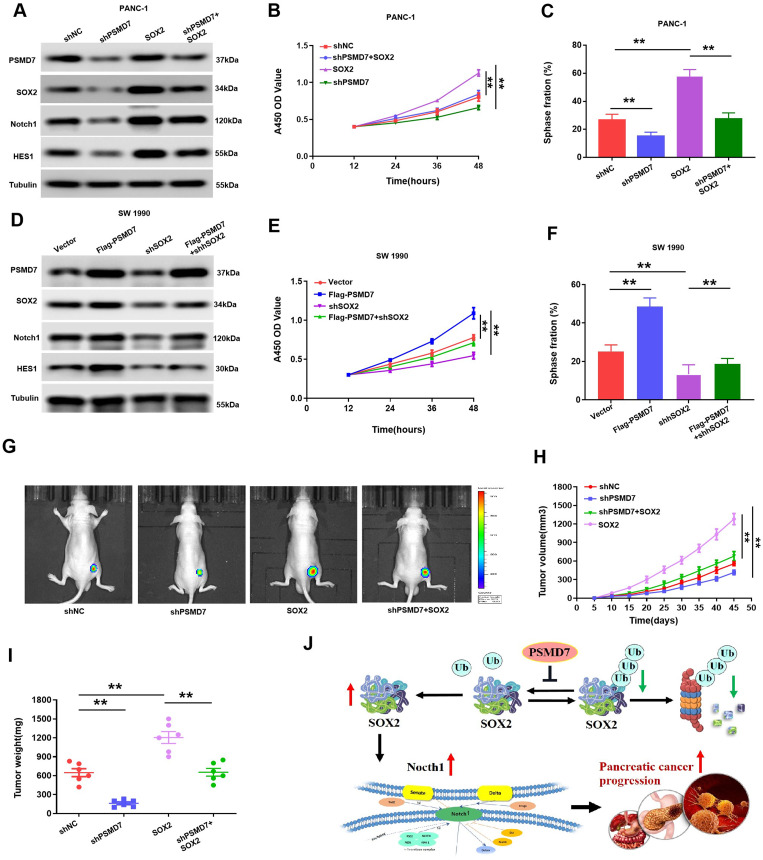
PSMD7 oncogenesis depends on the SOX2-Notch1 pathway. (**A)** Expression of HES1, Notch1, and PSMD7 in PC cells that were co-transfected with SOX2 and sh-PSMD7. (**B)** and **(C)** EdU and CCK-8 analysis of cell proliferation in the PANC-1 cells that express shPSMD7 or shNC, with or without overexpression of SOX2. (**D)** Expression of HES1, Notch1, and PSMD7 in PC cells that were co-transfected with sh-SOX2 and Flag-PSMD7. (**E)** and **(F)** EdU and CCK-8 analysis of cell proliferation in SW 1990 cells that express exogenous PSMD7 or control vector, with or without the transfection of sh-SOX2. (**G)** Mice injected with luciferase-expressing PANC-1/SOX2, PANC-1/shPSMD7, PANC-1/shNC, or PANC-1/shPSMD7 + SOX2 cells were examined through IVIS imaging system. (**H)** Tumour volumes in the PANC-1/SOX2, PANC-1/shPSMD7, PANC-1/shNC, or PANC-1/shPSMD7 + SOX2 group. Volumes of tumours are expressed as the mean ± SD, *n* = 6, **p < 0.01. (**I)** Tumour weight in the PANC-1/SOX2, PANC-1/shPSMD7, PANC-1/shNC, or PANC-1/shPSMD7 + SOX2 group. *n* = 6. **p < 0.01. (**J)** Suggested mechanistic scheme for PSMD7 to facilitate the Notch1 signalling pathway mediated by SOX2 in PC, *r* = 0.3649, *p* = 0.0067. *p < 0.05, **p < 0.01

Finally, rescue experiments were conducted to investigate the dependence of PSMD7 oncogenic effect on SOX2 stabilization in PC. As shown in Fig. 8A-C, the knockdown of PSMD7 dramatically diminished the proliferative capacity of PC cells, while simultaneous SOX2 overexpression diminished this capacity. Likewise, augmentation with SOX2 rescued PSMD7 knockdown-induced PC cell proliferation (Fig. 8D-F). Simultaneously, we injected PANC-1 cells stably expressing sh-NC, sh-PSMD7, SOX2 or sh-PSMD7 + SOX2 into the subcutaneous part of the leg of nude mice, and tumour growth was monitored. As shown in Fig. 8G-I, the tumor volume and weight of mice harboring PSMD7-silenced cells exhibited a remarkable reduction, whereas simultaneous SOX2 overexpression completely abrogated the antitumour effects of PSMD7 knockdown. Consequently, the deubiquitylase PSMD7 activated the Notch1 pathway via altered degradation of SOX2, thereby promoting PC progression (Fig. 8H).

## Discussion

Ubiquitination is an essential post-translational modification of proteins that exerts significant influence on cellular differentiation, proliferation, and apoptosis regulation [25]. Previous investigations have revealed that the ubiquitin ligase FBXW7 modulates epithelial-mesenchymal transition and tumour metastasis [26]. Likewise, the deubiquitination process through the stabilisation of flagellin and hydrolysis of ubiquitin has attracted increasing attention [12]. Proteasome inhibitors have recently demonstrated remarkable anticancer activity [27–29]. Proteasome 26 S subunit non-ATPase 7 (PSMD7) was first discovered as a non-ATP subunit of 26 S proteasome, also known as Mov34. It is located on the 19s regulatory granule of proteasome and belongs to the deubiquitinating enzymes (DUBs) JAMM domain metalloproteinase family [30, 31]. PSMD7 and PSMD14 are closely linked to the central components of the 26 S proteasome. There is an interaction between PSMD7 and PSMD14 that activates proteasome function and modulates the degradation of ubiquitinated substrates. Numerous studies have consistently suggested PSMD14 as a promising target for anti-proteasome therapy in diverse cancer types [9, 12, 32]. Our study indicates that variations in the expression of PSMD7 are strongly correlated with PC proliferative ability. Therefore, PSMD7 may be an underlying target for PC therapy. Recent reports have revealed that PSMD7 aberrant expression in breast and lung cancer tissues is associated with poor prognosis [10, 12]. These results agree with those of the present study. Although the latent mechanisms of action of PSMD7 in patients with PC may be diverse and complex, PMSD7 remains an essential gene involved in oncogenesis and tumour progression. In this study, we demonstrated a significantly higher expression of PSMD7 in PC tissues compared to adjacent tissues. Knockdown or overexpression of PSMD7 could influence PC cell proliferation. Hence, PSMD7 dysfunction may be among the factors influencing PC progression.

Notch1 signalling is a versatile and evolutionarily conserved pathway that participates in tumour progression and carcinogenesis in various cancers [33–36]. SOX2 plays a pivotal role in the Notch1 signaling pathway and exhibits robust regulation at three distinct levels: transcriptional activity, subcellular localisation, and protein stability [16, 17, 37]. SOX2, a transcription factor encoded by TCF8, combines with the E-box sequence CAGGTG/A to modulate gene expression [38]. Previous studies have emphasised the repression of target genes by SOX2. However, there is growing evidence that SOX2 activates the transcription of target genes [39]. SOX2 is strongly associated with the genesis and progression of various tumours. In prostate cancer, SOX2 overexpression has emerged as a critical marker for evaluating metastasis [40]. In breast cancer (BC), SOX2 contributes to tumour malignancy and causes cells to display stromal-like features [41]. Recent studies have reported that SOX2 induces resistance to radiotherapy in BC, which plays a pivotal role in the emergence of chemotherapy resistance in tumor cells [17, 42]. In this study, it is noteworthy that SOX2 expression was affected in cells treated with PSMD7 knockdown and overexpression. In the cells, SOX2 directly interacts with PSMD7. Previous studies have found that PSMD7 is highly expressed in esophageal squamous cell carcinoma cells, and knockdown of PSMD7 leads to a decrease in proteasom activity and the ability of recognition to poly-ubiquitinated chain and cleavage of the protein from ubiquitinated chain, which inhibits the mTOR/p70S6K pathway and the proliferation of esophageal squamous cell carcinoma cells [13]. Moreover, PSMD7 can enhance the deubitization of RAD23 homolog B (RAD23B) protein, reduce the degradation of RAD23B, and promote the proliferation, invasion and cisplatin resistance of gastric cancer cells [9]. In this study, we found that knockdown of PSMD7 decreased the activity of proteasome by affecting the function of 19s proteasome. Meanwhile, PSMD7 knockdown decreased the ability to remove the ubiquitination of SOX2, resulting in an increase in the ubiquitination of SOX. On the contrary, PSMD7 overexpression could significantly increase the activity of proteasome. PSMD7 overexpression enhanced its deubiquitination function, and further reduced the ubiquitination of SOX2. Therefore, we concluded that PSMD7 affected the degradation pathway of SOX2 protein via deubiquitination.

## Conclusions

Taken together, our findings collectively demonstrated a conspicuous upregulation of PSMD7 levels in PC tissues and were strongly correlated with clinical characteristics. Higher PSMD7 levels are correlated with disease progression and serve as a prognostic indicator for reduced survival rates in patients with PC. PSMD7 knockdown suppresses PC cell proliferation. Considering these outcomes, PSMD7 may act as a promising prognostic biomarker for patients with PC and be regarded as an underlying target for future PC therapy.

### Electronic supplementary material

Below is the link to the electronic supplementary material.

Supplementary Material 1

Supplementary Material 2

Supplementary Material 3

## Data Availability

All data and materials supporting these conclusions are included in the main paper.
