# Supplementary material for: Deubiquitinase PSMD7 facilitates pancreatic cancer progression through activating Nocth1 pathway via modifying SOX2 degradation
Source: Cell Biosci. 2024 Mar 17;14:35. doi: 10.1186/s13578-024-01213-9 (PMC10944620; doi:10.1186/s13578-024-01213-9)
Supplement: Supplementary file 3 — Supplementary Material 3 [file 13578_2024_1213_MOESM3_ESM.docx]

**Supplementary Figure legends**


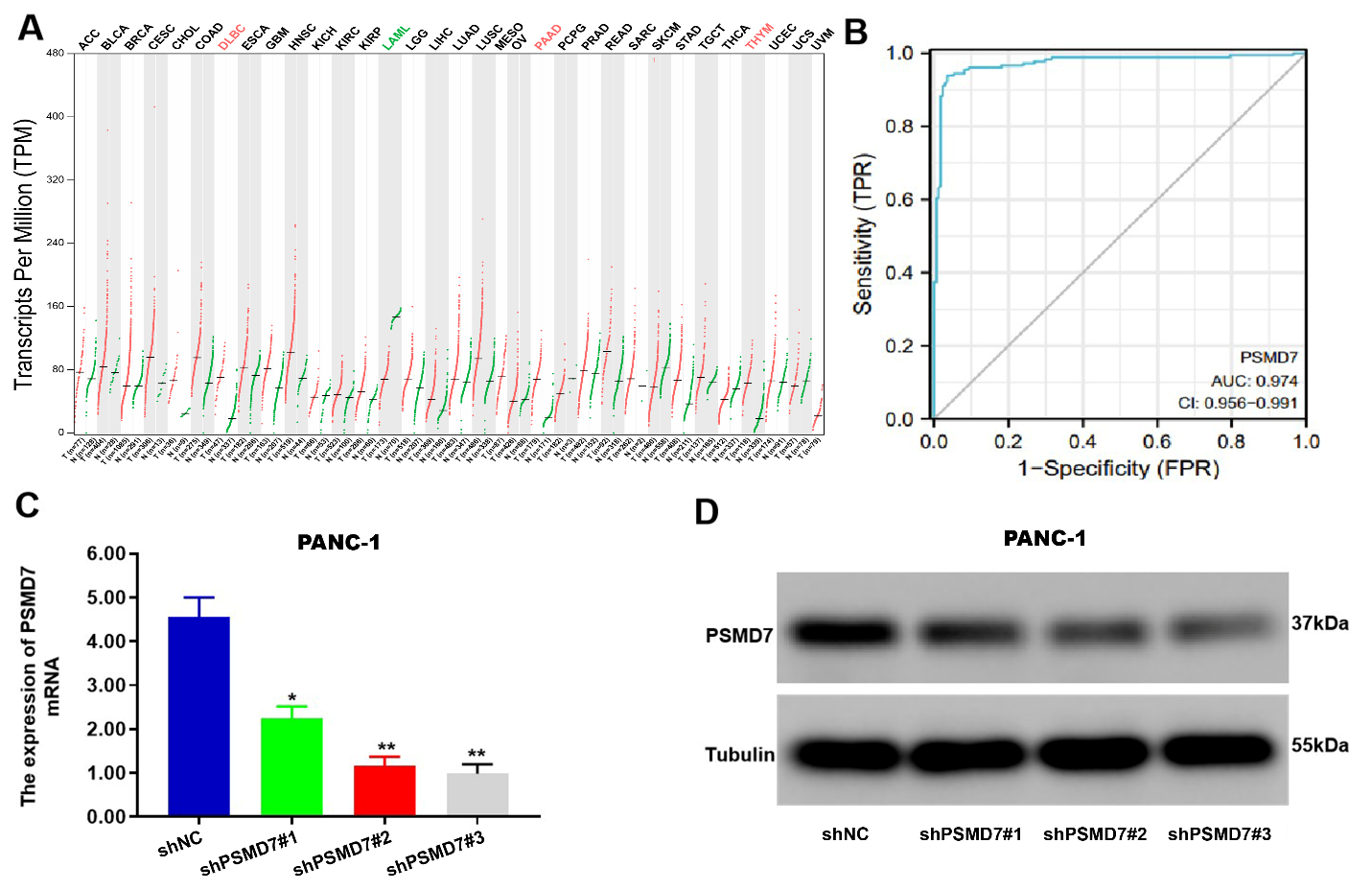


**Supplementary Fig. 1. Expression and diagnostic efficacy of PSMD7.**

**A,** Presentation of PSMD7 expression in pancreatic cancer using GEPIA online tool (red represents high expression in tumour, green represents high expression in normal tissue, and both red and green are statistically different). **B,** ROC curve was exploited to assess the PSMD7 diagnostic performance against pancreatic adenocarcinoma (FPR, false positive rate; TPR, true positive rate). **C and D,** PSMD7 mRNA long with protein levels were explored via qRT-PCR and western blotting in PANC-1 cells that were transfected with shPSMD7 or shNC. Tubulin was applied as a loading control. Data present the mean ± SD of triplicate assays and were analysed statistically via Student's t-test; **p* < 0.05, ***p* < 0.01.


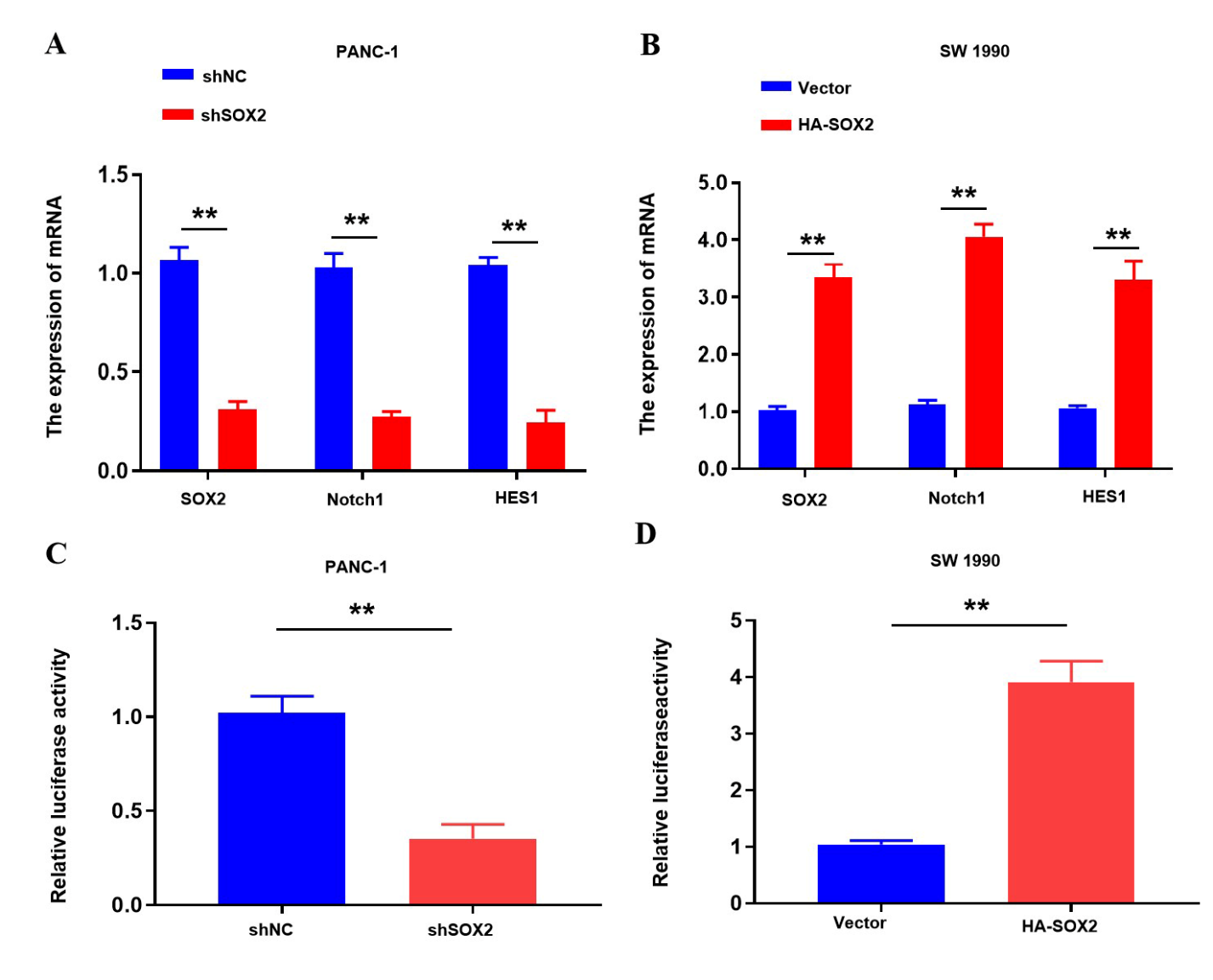


**Supplementary Fig. 2. SOX2 positively regulates the NOTCH1 pathway.**

A **and B,** The HES1 and Notch1 mRNA expression levels were assayed by qRT-PCR with SXO2 knockdown in PANC-1 cells or overexpression in SW 1990 cells . C**,** Luciferase activity of the NOTCH1 pathway-reporter in shNC or shSOX2 transfected PANC-1cells. **D,** Luciferase activity of the NOTCH1 pathway-reporter in vector or HA-SOX2 transfected SW 1990 cells. Data are obtained from three independent biological replicates and are presented as mean ± SD. **p* < 0.05, ***p* < 0.01, as indicated.
